# Our Friends beyond

**Published:** 1881-07

**Authors:** 


### OUR FRIENDS BEYOND.

WHAT are they? What are we to them? What are they to us? Shall we know them when we get there? There are no questions that so deeply interest us as these. At the same time there are none on which we are so ignorant. Most people think of death as something radical in its effects, something that will remove all former trace of ourselves, leaving nothing of our personality.

That which makes you worthy or the love, confidence and compansionship of friends, will remain the same. What there was which made that friend, gone before, dear to you, and which you still cherish in memory, is what makes the friend a glorified saint; she is to you, save in bodily presence, all that she ever was.

To illustrate: A member of your family has some disease which makes it necessary for him to seek a change of climate. He goes to the sunny land of Italy. There he regains his health; but, fearing to return to this rugged climate, remains there. What is that friend to you—save for an occasional letter—more than if he were in the Beyond, and lived only in your memory? You will never see his face, nor hear his voice, and he thinks of you as a friend left behind.

So we should think of departed ones. Their appearance is changed; they have the "celestial body." Their speech is changed; they use spiritual language. There is a great change: but is it not like the change of a diseased man restored to perfect health? The change is in the perfect restoration of the soul to a state of innocence and holiness. Yet in every essential it is the same. The sunny land of Heaven may be farther away to the senses than the sunny land of Italy; yet Heaven is as real, its inhabitants are as real, in thought, feeling, form and relation to one another as are people familiar to each other on earth.—*Golden Rule.*

